# Food, Eating, and the Gastrointestinal Tract

**DOI:** 10.3390/nu12040986

**Published:** 2020-04-02

**Authors:** Dan M Livovsky, Teorora Pribic, Fernando Azpiroz

**Affiliations:** 1 Digestive Diseases Institute, Shaare Zedek Medical Center, Hebrew University of Jerusalem, 9103102 Jerusalem, Israel; danlivo@yahoo.com; 2 Digestive System Research Unit, University Hospital Vall d’Hebron, Passeig de la Vall d’Hebron 119, 08035 Barcelona, Spain; teodora.pribic@gmail.com; 3 Centro de Investigación Biomédica en Red de Enfermedades Hepáticas y Digestivas (Ciberehd), Av. Monforte de Lemos 3-5, 28029 Madrid, Spain; 4 Departament de Medicina, Universitat Autònoma de Barcelona, 08193 Cerdanyola del Vallès, Spain

**Keywords:** food ingestion, digestion, satiety, digestive well-being

## Abstract

Food ingestion induces a metered response of the digestive system. Initially, the upper digestive system reacts to process and extract meal substrates. Later, meal residues not absorbed in the small bowel, pass into the colon and activate the metabolism of resident microbiota. Food consumption also induces sensations that arise before ingestion (e.g., anticipatory reward), during ingestion (e.g., gustation), and most importantly, after the meal (i.e., the postprandial experience). The postprandial experience involves homeostatic sensations (satiety, fullness) with a hedonic dimension (digestive well-being, mood). The factors that determine the postprandial experience are poorly understood, despite their potential role in personalized diets and healthy eating habits. Current data suggest that the characteristics of the meal (amount, palatability, composition), the activity of the digestive system (suited processing), and the receptivity of the eater (influenced by multiple conditioning factors) may be important in this context.

## 1. Introduction

The importance of a healthy diet is well recognized, but to become acceptable, a diet needs to be attractive and gratifying. In this regard, it is crucial to understand the factors that determine the responses to ingested food and in particular the lasting effects following ingestion, i.e., the postprandial experience. In this context, the Nutrients Special Issue *entitled Food and Diet for Gut Function and Dysfunction* focuses on the role of the responses of the digestive system to food ingestion in normal conditions and in the disease state. The aim of the present paper is to provide an introductory overview to the Special Issue, outlining the effects of food ingestion on the brain-gut axis, i.e., the relations between digestive responses and the sensory experience. Purposely, this review is sketchy and descriptive; most of the experimental factual/information is provided in the different articles that compose the Special Issue. A comprehensive review on this subject, analyzing the relations between gastronomy and neurogastroenterology, has been previously published [1].

## 2. Physiological Responses to Meal Ingestion

During fasting, the gastrointestinal tract exerts a cyclic activity, alternating between periods of quiescence and periods of intense motor and secretory activity. The function of this stereotyped pattern, known as the migrating motor complex (MMC), appears to be the propulsion of residues from the lumen of the small intestine into the colon; thus, it is considered to be the intestinal housekeeper [2].

**Figure 1:**
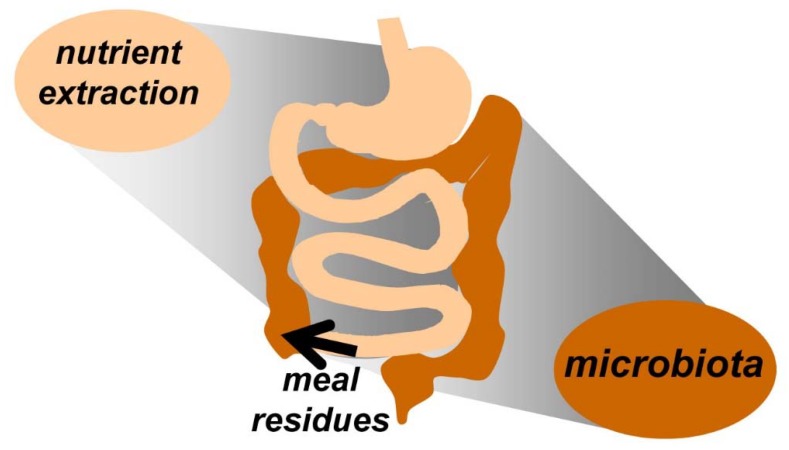
Digestive response to ingestion. The upper digestive system extracts meal substrates by a process of digestion and absorption. Non-absorbed meal residues pass into the colon and feed the microbiota.

With the initiation of a meal, the digestive system is stimulated leading to the suppression of the interdigestive motor pattern and the activation of the digestive process. The digestive process involves three semi-sequential phases that overlap over time: cephalic, luminal, and post-absorptive. The cephalic phase refers to events before and during the ingestion period. Indeed, even before ingestion, the digestive system starts with a series of preparatory procedures, associated in normal conditions with an anticipatory reward sensation, e.g., anticipation of a desired meal stimulates salivary and gastric secretion. Food ingestion and swallowing activate oropharyngeal and oesophagogastric responses (salivation, oesophageal peristalsis, and gastric receptive relaxation). The walls of the stomach are contracted and virtually collapsed during fasting and meal arrival into the stomach induces an active relaxation (gastric accommodation). Solid particles activate the antral pump with peristaltic activity, which starts a grinding process that transforms the meal into a liquid chime. A gradual re-contraction of the stomach during the postprandial phase pushes the chime into the small bowel [3]. The activity of the stomach and small bowel adapts to the requirements of the digestive process, so that, food is digested and subsequently absorbed by a sequence of complex physical and chemical processes that begin in the mouth and extend to the terminal ileum. Ultimately, non-absorbed residues reach the colon [4]; these dietary residues serve as substrates for gut microbiota, and in return, the gut microbiota can affect host physiology and digestive function (Figure 1).

Indeed, the large majority of human microbiota inhabit the colon, which provides a dedicated niche for this population of symbiotic organisms. Microbiota fermentation of meal residues releases a series of metabolites that, in turn, serve as substrates for other subsets of microbiota. Hence, the colon contains a biomass formed by microbiota, meal residues, and secondary metabolic products in a dynamic chain of metabolic reactions. The total volume of colonic biomass consists of approximately 500–800 mL with a 30% daily turnover (100–200 mL daily fecal output) [5]. Microbiota metabolism of some meal residues releases gas; hence, colonic gas production reflects microbiota metabolic activity [6]. Approximately 100 min after ingestion, unabsorbed residues start arriving into the colon, and gas production increases. The plateau lasts approximately 4–6 h depending on the meal composition and then gradually declines [7], but the effect persists as long as substrates remain available, such that the residue loads of consecutive meals contribute to gas production [8]. The volume of gas produced within 6 h after a meal ranges from 200 mL with a standard breakfast to 600 mL with a flatulogenic meal [7].

The activity of the muscles of the abdominal and thoracic walls depends on its content. Specifically, an increase in intraabdominal volume induces an adaptive relaxation of the diaphragm, which allows orad expansion of the abdominal cavity with compensatory contraction of the anterior abdominal wall; this somatic response prevents an increment in abdominal girth. The same adaptive response is triggered by ingestion of a meal, a phenomenon termed abdominal accommodation [9]. The extent of accommodation depends on the volume load. This somatic reflex has clinical relevance, because impaired abdominal accommodation produces abdominal distension, a frequent complaint in clinical practice; in these patients, an abnormal contraction of the diaphragm pushes abdominal content with protrusion of the anterior abdominal wall [10]. Physiologic changes in blood pressure, heart rate, blood flow in the superior mesenteric artery, and mesenteric vascular resistance, as well as in thermogenesis are also induced during the luminal and post-absorptive phase of digestion, but the specific meal-derived signals that control these responses are incompletely understood [11].

## 3. Food Ingestion and the Brain–Gut Axis

The entire digestive-absorptive process is finely regulated by a complex net of neuro-humoral feedback mechanisms, by which the gut is able to sense and react to intraluminal stimuli [4]. These reflex pathways are distributed within the autonomic and the enteric nervous system. This physiological configuration allows the gut to be highly versatile and adaptable. Stimuli in the gut may also activate afferent brain pathways, so that in addition to the digestive responses, meal consumption also induces sensory experiences that influence the control of food consumption and homeostasis (e.g., fullness and satiety) [12,13,14,15,16,17]. These sensory experiences are associated with pleasant or occasionally unpleasant sensations (changes in mood and digestive well-being), i.e., the hedonic dimension of the sensory experience [12,13,14,18,19]. Changes in the activity of the central nervous system (CNS) in response to meal ingestion have been documented by means of functional brain imaging (e.g., functional magnetic resonance imaging and positron emission tomography) [20,21,22,23,24,25,26]. These studies reveal the crucial role of the CNS in the control of food intake and the conscious perception of sensation and in the maintenance of homeostasis [27]. Meal ingestion is associated with changes in the blood levels of several compounds [13,28,29,30]. These compounds may be derived directly from the food (e.g., amino acids, lipids, and glucose), produced by the organism in response to the meal (e.g., hormones and neuropeptides) or may be the result of the metabolism of non-absorbed residues by the colonic microbiota [31,32]. The bidirectional interaction between the mind and the digestive system, involving both neural and humoral pathways is known as the brain–gut (or gut–brain) axis. There is a dynamic cross-talk between host and microbiota, the messengers and circuits are poorly understood, but metabolites derived from microbiota activity may play a role [33]. Attending to the role of microbiota, the concept has been extended to encompass the microbiota–gut–brain axis.

## 4. Sensations before and during Food Ingestion

The biological response to food is complex and involves events before, during, and after the meal. Indeed, the sensory experience related to meals starts before the ingestion period [34]. The anticipatory experience before the meal depends on factors related to the meal (e.g., meal appearance, smell), as well as the subject’s homeostatic status (e.g., degree of hunger) and cognitive state (e.g., expectations).

Appetite is an imprecise term, since it may refer to differing concepts. It can be used as an all-inclusive term to cover all aspects of food intake, preference, motivation, and selection, or as a reference to the sensory and qualitative aspects of eating and the responsiveness to environmental stimulation [16]. Thus, the concept of “meal wanting” was coined in order to describe the pre-ingestive response to a specific food, i.e., the desire to eat that particular product [35,36].

The motivational aspect of food consumption has been the subject of numerous studies [18,37,38,39,40]. The homeostatic regulation of eating depends on the degree of hunger (or satiation). Food ingestion reduces hunger sensation and depending on the amount ingested, induces satiation and fullness sensations; in principle satiation is a signal to stop ingestion. Remarkably, satiation is taste-specific, thus food diversity increases meal consumption. In any case, with increasing satiety and fullness, “meal wanting” and the desire to eat a food of choice decrease. The homeostatic regulation of food ingestion might be overruled by other factors such as the hedonic drive; this may lead to excessive eating and it has been associated with obesity and eating disorders [41,42]. Cognitive factors and the habits of the subject also play an important role. These include memories, beliefs, expectations, and thoughts related to what the subject considers about (a) the characteristics of the meal (satiating capacity, quality, health properties); (b) meal availability, either present and/or in the near future; (c) what the eater believes is beneficial or necessary at a certain moment (e.g., low-fat food for an obese patient). Notwithstanding, a decisive factor on food consumption is, at the end, what is present in the menu or in the plate, and thus, the importance of the serving portions in food consumption.

The most important aspects of food that can be detected by the senses (organoleptic properties) are taste, smell, and texture, although other properties, such as temperature, sound, and appearance, are also involved. The biology and physiologic mechanisms of food sensation have been thoroughly studied [43,44,45,46]. For the purpose of this review, three concepts in this regard will be considered: taste, flavor, and palatability

### 4.1. Taste

The taste perception, or in other words gustation, is triggered by the stimulation of specific receptors in the mouth and in the pharynx by molecules in a liquid environment. The five tastes that are widely accepted to play a major role in the experience of sensing food are: salty, sour, umami, bitter, and sweet. However, many additional tastes (~20) including fatty acid, metallic, and electric have been proposed as candidates [47,48]. Nevertheless, the specific receptors involved in sensing each taste are still not fully understood. Moreover, the distribution and number of taste buds may differ in different persons as shown by the extreme sensitivity to bitter taste secondary to increased number of taste buds in up to 25% of the population, these individuals can be considered as supertasters [49,50]. Interestingly, taste receptors similar to those responsible for food sensing in the mouth have been identified along the gastrointestinal tract; their function appears to be related to regulation of gut function and homeostasis independent form taste sensation [3,51,52,53,54].

### 4.2. Flavor

Flavor is a complex and multi-modal sensory experience that occurs during food tasting [43] and directly involves gustatory and olfactory sensations [55]. However other senses, such as proprioception, temperature, vision, and sound, can impact flavor perception. Consequently, flavor is a combined interoceptive and exteroceptive experience.

The hot sensation in spices (pungency) is produced by capsaicin and other chemical components. These molecules are not sensed by taste receptors, but by sensory nerve endings analogous to those signaling pain, and the sensation is driven by the trigeminal nerves. Pungency is an important aspect in the flavor of food.

Olfaction, i.e., the perception of smell, deeply interacts with and enhances the perception of taste. The process of olfaction involves the orthonasal and retronasal systems. The former is activated by inhaling volatile compounds that enter the nose via the nostrils, while the latter is activated by volatile compounds released from the food during chewing and swallowing that reach the retronasal system through the posterior nares when the individual exhales. Sensory inputs form the anterior and posterior nasal systems are transmitted through different neural pathways to different brain areas. There are roughly 500 types of odorant receptors in the nasal mucosa [56,57,58]. Certain molecules activate several types of receptors, while each receptor type may be activated by different molecules. Thus, the precise odor of a product depends on the mixture of activated receptors.

Food temperature influences taste since it regulates the access of molecules from volatile compounds to sensing receptors. Moreover, in some individuals, sweet, sour, bitter, or salty sensations can be evoked by the application of heat to different parts of the tongue; a characteristic known as “thermal taster”.

Food texture is perceived by touch and proprioception and depends on the rheological properties of the meal (i.e., flow of matter/changes in matter in response to applied force). Important hints to the texture of the meal are obtained before ingestion by physical manipulation (cutting, touching, mixing), vision, and occasionally by sound. During the oral phase, the feel of the food (mouth feel) is sensed by mastication and tongue shearing. Also, the sound that food makes within the mouth (e.g., crispy fries or crunchy crackers) is important for flavor sensing. There is an almost endless combination of textures that can be present in a meal (e.g., elasticity, consistency, astringency, viscosity, granularity, smoothness, sogginess, etc.).

Food appearance is an important factor that shapes the expectations related to flavor. Indeed, in a very interesting study, experimental mismatch of color in fruit-flavored beverages and candies was associated with incorrect identification of the product, (e.g., a yellow candy tends to be recognized as lemon flavor regardless of the true taste) [59]. It should be noted that mastication, salivation, and tongue movement change the rheological properties of food and molecules activating taste and smell are widely spread in the oral and retronasal cavities. Hence, the oral phase of digestion modifies the interoceptive properties of food that determine flavor (taste, odor, texture, pungency, and temperature).

### 4.3. Palatability

Palatability it is not a characteristic of the food itself, but rather it refers to the hedonic sensation (pleasurable or aversive) derived from food tasting (i.e., how good the food is perceived) [60]. It depends on the organoleptic properties of the meal, but more importantly on the receptiveness of the eater: the state of the eater before the meal (e.g., hunger), flavor perception, and interpretation [61,62]. Consequently, the palatability of the meal is dynamic and it changes during ingestion (palatability decreases as hunger decays and satiation arises). Subject attentiveness also plays an important role, e.g., palatability is more pronounced when the subject is paying attention to the meal than when distracted. The notion of “meal liking”, in contrast to “meal wanting”, reflects the hedonic dimension of the gustatory experience, i.e., palatability, but it extends further to include the postprandial sensation of satisfaction and digestive well-being [35]. Previous experience and memory influence palatability, such that more palatability is associated to flavors that are congruent and recognized. Conversely, exposure to an unfamiliar or aversive flavor can translate into a decrease in palatability [46]; however, in special situations (e.g., dinner in a special restaurant) the receptivity of the subject is increased and an unrecognized, unexpected, or incongruent gustatory experience (e.g., cold soup, salty ice cream) may increase the palatability of the meal by the surprise factor.

## 5. The Postprandial Experience

**Figure 2:**
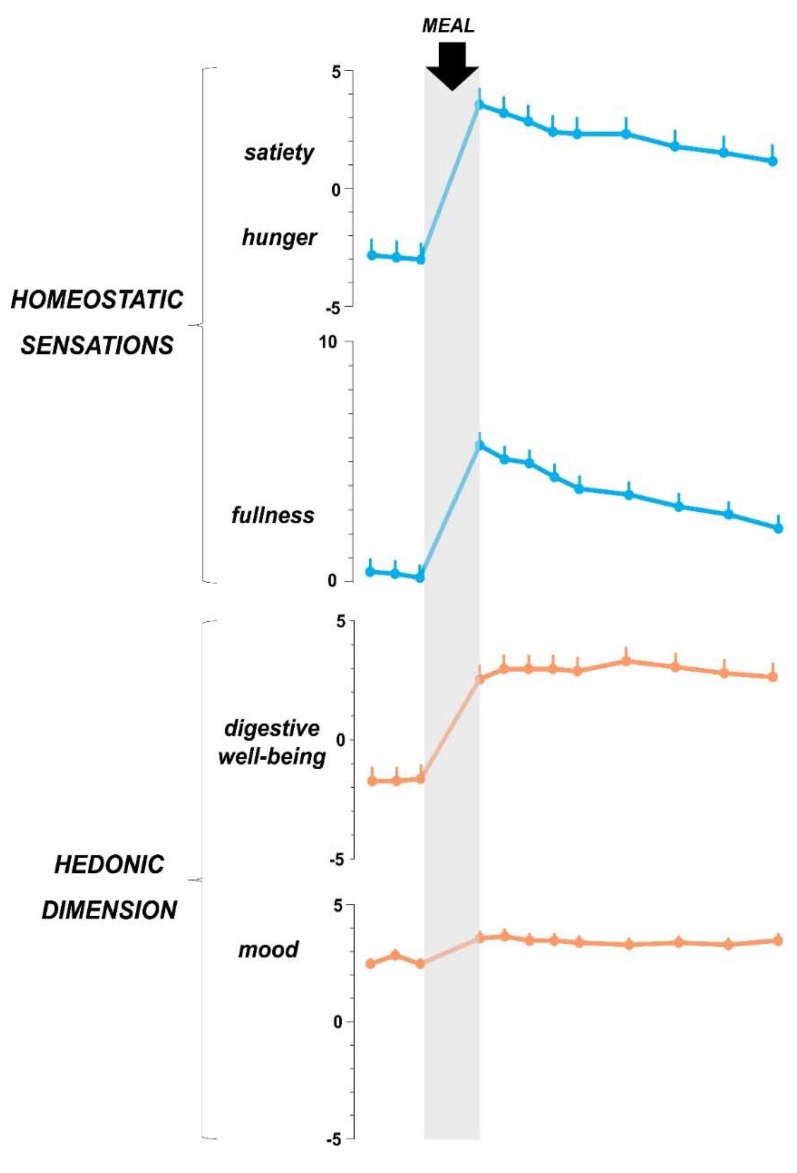
Sensations related to food ingestion. In healthy women (*n* = 12) a. palatable comfort meal induced homeostatic sensations (satiety, fullness) with pleasant hedonic dimension (increased digestive well-being and mood). Data from reference 79.

The sensations that arise during the time of ingestion extend into the postprandial period. Hence, the postprandial experience comprises homeostatic sensations (satiation, fullness) and hedonic sensations (i.e., post-prandial mood and digestive well-being) (Figure 2).

Homeostatic and hedonic sensations are not dependent on each other and experimental data in humans suggests that they are mediated by different mechanisms [27]. Indeed, when measured by analogue scales, hedonic and homeostatic responses, correlate with specific changes in brain activity and circulating metabolites [14,20,27]. Moreover, the hedonic response to postprandial fullness and/or satiety may be positive or negative in response to several conditions (see next section).

The postprandial experience is dynamic: hedonic and homeostatic sensations are more powerful in the immediate post-ingestion period and weaken progressively during the postprandial period. While, the term satiation refers to the sensation perceived during ingestion, the term satiety refers to the sensation perceived after the meal. Yet, the distinction between satiation and satiety is not completely supported by biological evidence and linguistically, this difference is not present in many languages. Satiation gradually decays during the postprandial state and regulates the inter-meal interval. Hence, by the end of the digestive process, subjects experience hunger again as a physiologic sign for the next meal. The type and amount of food that were consumed will have an important influence in the rate of decline in satiety and subsequently reappearance of hunger sensation. Furthermore, throughout the digestive process, additional sensations secondary to autonomic activity, e.g., warmth and sleepiness, appear. One to two hours after the meal, when undigested residues arrive into the colon and are fermented by the microbiota, sensations related to gas, such as flatulence and borborygmi, may arise, particularly after high flatulogenic meal (meals rich in non-absorbable, fermentable residues).

**Figure 3:**
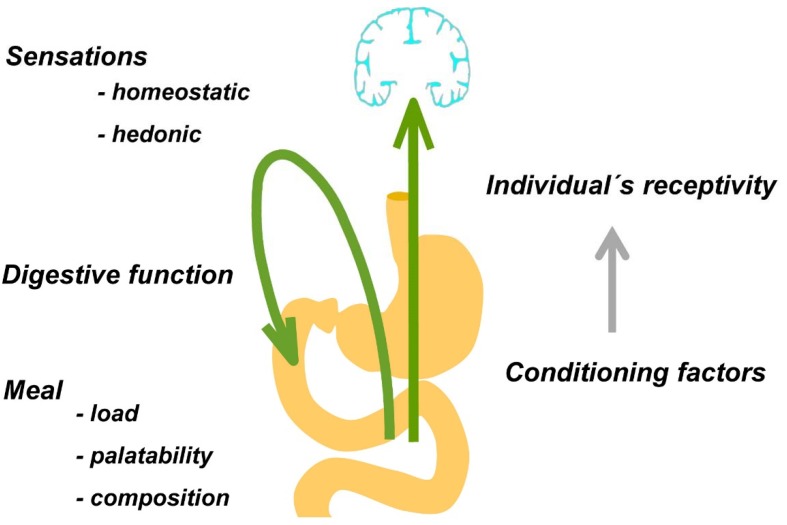
Biological responses to food ingestion. Meal ingestion induces digestive and sensory responses. Homeostatic (satiety, fullness) and hedonic sensations (digestive well-being and mood) depend on the characteristics of the meal, the digestive response, and the individual’s receptivity, which can be influenced by multiple conditioning factors. Adapted from reference [1].

The postprandial experience depends on interacting factors related to (a) the digestive function, (b) the characteristics of the meal, and (c) the individual’s responsiveness (Table 1 and Figure 3).

### 5.1. Digestive Function

The leading determinant of digestive sensations is the digestive function, so that a satisfactory postprandial experience depends on adequate digestive responses to a meal [15,17,69,70]. For example, this has been shown by disturbing gastric accommodation: inflation of a gastric balloon impairs the postprandial sensations [12]. Remarkably, patients with functional gut disorders show intestinal dysfunction and hypersensitivity so that the postprandial period after normal meals turns symptomatic. Nevertheless, the mechanism and specific food components that are responsible for the arousal of symptoms in these patients, or occasionally in healthy individuals, are incompletely understood. In healthy individuals, fat has a strong influence on digestive function; it induces satiety and fullness and enhances gut sensitivity. This response is augmented in patients with functional gut disorders and is related to their intolerance to fat ingestion [71,72]. Indeed, in functional dyspepsia and irritable bowel syndrome, fatty foods are the most common features of the meal that trigger symptoms [71,72].

### 5.2. Characteristics of the Meal

Many properties of the meal may influence the postprandial experience. Nevertheless, all are related to the meal load (i.e., amount of ingested food), palatability, and composition.

#### 5.2.1. Meal Load

Obviously, homeostatic sensations depend on the amount of food consumed, with larger meals inducing more satiety and fullness. However, the relation of homeostatic and hedonic responses to a meal is bimodal depending on the meal load. Ingestion of a gratifying meal induces both satiety and satisfaction, i.e., homeostatic and hedonic sensations increase in parallel. Nevertheless, this is true up to a certain limit when the relation overturns and increasing meal loads still increase satiety but with a gradual decrease in digestive well-being; the nadir is reached with large meals at the point of full satiation, which induces a uncomfortable fullness sensation [63].

#### 5.2.2. Meal Palatability

The central role of palatability in the postprandial experience was elegantly demonstrated by a recent study. Two meal courses where carefully prepared to have the same texture, consistency, temperature, and color, but with different palatability. The first one was a potato and cream cheese course and the second a vanilla cream dessert. Based on these courses, two different meals were prepared: a conventional meal was served by giving the first course as main dish and then the second as a dessert; an unconventional meal was served by mixing the two courses in a single dish, preserving the same physical characteristics of the two individual components. Indeed, both meals had the same composition and physical characteristics. Twenty-two healthy males were randomized in a cross-over design to receive the conventional meal and the unconventional meal on different days. The conventional meal was found palatable and induced a pleasant sensation. By contrast, participants found the unconventional meal unpalatable and reported higher fullness sensation with lower mood and digestive well-being [64].

#### 5.2.3. Meal Composition

Despite the central role of palatability on the postprandial experience, other factors are also important. Indeed, appetizing and desirable meals may be followed by a negative, even symptomatic postprandial experience. Thus, in addition to palatably, some meal components may have an intrinsic effect in postprandial sensations and may produce symptoms by themselves. Therefore, the gustatory experience during ingestion (i.e., food taste and palatability) cannot forecast its postprandial effects, and even delicious meals may be followed by negative postprandial sensations.

The specific effects of particular meal components on postprandial sensations have not been properly described. Still, fat is the best described food component related to postprandial sensations. On the one hand, fatty “comfort” food in healthy individuals is associated with comfort and positive mood (reviewed in [19]); on the other hand, after a certain threshold, fat in excess decreases satisfaction and induces aversive sensation in the postprandial period [14,65]. It is plausible that intraluminal and post-absorptive mechanisms are behind the effects of meal composition on postprandial sensations. The influence of meal composition and meal-related signals on digestive function and perception has been specifically addressed in another paper of this Special Issue [73].

### 5.3. The Individual’s Receptivity

The receptivity of the individual to the meal is determined by constitutive and inducible factors. In the first place, among constitutive factors, innate qualities are key to the postprandial experience. Recent proof-of-concept studies indicate that sex may play an important role, and showed that the response to the same meal is different in women and men. These studies showed that women enjoy and tolerate smaller meals than men, i.e., a meal that induces satisfactory satiety in men may induce aversive fullness sensation in women [63,66,74]. Conceivably, other constitutive characteristics may also play a role in the individual predisposition to respond to a meal, so that some individuals may be more gifted to appreciate and enjoy meals.

The receptivity of individuals depends also on inducible factors. Indeed, multiple conditioning factors may influence the postprandial experience. For instance, some studies have shown the role of homeostatic conditions, e.g., less hunger before ingestion results in more postprandial satiety and less satisfaction [67]. The habits of the individual are also important and, independently of hunger, influence the response to a meal: a meal consumed at an unconventional schedule induces more satiation and less satisfaction than at the right time, and interestingly, women are more susceptible to the eating schedule than men [66]. A study showed that cognitive/sensory conditioning by an educational intervention influences the postprandial experience: hedonic and homeostatic responses to the test meal, were both enhanced by the intervention [68], i.e., by an educational intervention the subjects learned to enjoy a test meal and experienced more satiation. The potential role of education in the modulation of the postprandial response may have important applications in different areas, including food consumption, acquisition of healthy habits, and in the treatment of digestive symptoms. Conceivably, the diner’s status may be also influenced by a large variety of environmental factors.

## 6. Conclusions

The data presented in this review indicate, first, that the postprandial experience is a key aspect of the biological response to food ingestion, and second, that a number of factors may determine this response. These factors are related to the characteristics of food (specifically, amount, palatability, and composition), the function of the digestion system (e.g., an impaired digestive response hampers the postprandial experience), and the responsiveness of the eater, that depends on constitutive (e.g., sex) and inducible factors; the latter may be influenced by a myriad of conditioning stimuli. In order to enhance the gastronomic experience, not only the food, but also the eater requires preparation. Investigations on the role of education and conditioning in shaping the individual’s receptivity may be the key to the development of healthy eating habits and the design of personalized diets.

## Figures and Tables

**Table 1:** Factors that influence the postprandial experience: previous studies.

Reference	Design and Outcomes	Aim	Participants	Interventions	Main Results	Conclusions
**Malagelada et al., 2015** [12]	Randomized, crossover trial Responses to test meal (a) sensations by scales (b) gastric tone by barostat	Effect of digestive function on perception	Healthy volunteers: 25 women 17 men	Distortion of digestive function by gastric distention or duodenal lipids	Experimental distortion of digestive function affects independently homeostatic and hedonic sensations after a meal	**The digestive function determines the postprandial experience; homeostatic and hedonic sensations are independent**
**Malagelada et al., 2016** [13]	Open label study Responses to test meal (a) sensations by scales (b) metabolomic analysis	Metabolomic biomarkers of postprandial sensations	Healthy volunteers: 9 women 9 men	Ingestion of a test meal at the rate of 50 mL/min at until maximum satiation	(a) satiation correlated with increase in glucose and valine; (b) well-being and decrease in choice eating correlated with increase in triglycerides; (c) abdominal discomfort inversely correlated with increase in lipids	**Postprandial sensations correlate with changes in circulating metabolites**
**Pribic et al., 2017** [20]	Open label study Responses to probe meal (a) sensations by scales (b) fMRI scans before and after probe meal	Brain networks related to postprandial sensations	38 healthy males	Probe meal on two days with and without fMRI	(a) sensations were similar with and without fMRI; (b) Ingestion was associated with increase in thalamo-cortical connectivity and decrease in insular-cortical connectivity; (c) a larger decrease in insular-anterior cingulate cortex connectivity and was associated with higher satiety, fullness, and digestive well-being	**Postprandial sensations correlate with changes in brain connectivity functional networks**
**DuBose et al., 1980** [59]	Open label study Identification of flavor of test foods	Influence of food color on flavor perception	Healthy volunteers	Test foods with colorants and flavorants: (a) masking of color; (b) color-flavor incongruence (e.g., green colored -orange flavor).	Color masking or distortion impaired flavor identification	**Flavor perception is influenced by color of food**
**Monrroy et al., 2019** [63]	Randomized parallel trial. Sensations in response to comfort meal by scales	Role of gender on the responses to a comfort meal	Healthy volunteers: 10 women 10 men	Comfort meal ingested stepwise until full satiation	In women the meal loads required to achieve maximal satisfaction and full satiation were smaller than in men. Hence women enjoyed and tolerated smaller meal loads than men	**Gender is a constitutive factor that determines the meal experience**
**Pribic et al., 2018** [64]	Randomized crossover trial. Sensations in response to test meals by scales	Effect of palatability on postprandial sensations	22 healthy men	2 meals with identical composition and physical characteristics but different palatability: (a) conventional (potato cream followed by vanilla cream); (b) unconventional meal (mixture of both creams).	The unconventional was found less palatable and meal produced more fullness and less satisfaction than the conventional meal	**Food palatability bears a direct relation to hedonic but inverse relation to homeostatic sensations.**
**Pribic et al., 2018** [65]	Randomized crossover trial. Sensations in response to test meals by scales	Influence of meal composition independently of palatability on postprandial sensations	12 healthy men	2 meals with the same physical and organoleptic characteristics (taste, smell, texture, color, and temperature) but different composition: (a) low-fat; (b) high-fat test meal	While palatability was similar, the high-fat mal induced more satisfaction than the low-fat meal, without significant differences in homeostatic sensations	**Meal composition determines the postprandial experience independently of palatability.**
**Masihy et al., 2019** [66]	Randomized parallel trial. Responses to probe meal: (a) sensations by scales (b) physiological measures	Influence of eating schedule on postprandial responses: gender effects	Healthy volunteers: 10 women 10 men	Lunch-type meal eaten at: (a) habitual afternoon schedule; (b) unconventional morning schedule	No schedule effect on physiological responses to probe meal in women and men were detected. However, in contrast to men, in women, the probe meal at unconventional time induced less satisfaction than at the conventional time	**Women are more susceptible to the influence of eating schedule on the postprandial experience than men.**
**Pribic et al., 2017** [67]	Randomized cross over. Sensations in response to test meals by scales	Influence of appetite on postprandial experience	12 healthy men	Probe meal consumed two hours after: (a) low-calorie breakfast; (b) high-calorie breakfast	As compared to the low-calorie breakfast, with the high-calorie breakfast subjects were less hungry before the probe meal and experienced more postprandial fullness and less satisfaction	**Appetite before the meal influences the postprandial experience**
**Pribic et al., 2018** [68]	Randomized, parallel study. Sensations in response to probe meal by scales.	Influence of education on postprandial experience	Healthy men: 14 per group	Administration of probe meal on 2 days without and with prior educational intervention. One group received a sensory-cognitive intervention and the other a sham intervention	The sensory-cognitive intervention enhanced homeostatic and hedonic sensations after the probe meal, whereas the sham intervention had no effect	**The receptiveness of the subject and the postprandial experience can be conditioned by education**
